# Prise en charge chirurgicale à ciel ouvert de l’instabilité luno-triquétrale

**DOI:** 10.11604/pamj.2016.24.273.7898

**Published:** 2016-07-27

**Authors:** Badr Ennaciri, Emmanuel Beaudouin

**Affiliations:** 1 Department of Orthopaedics, Avicenna University Hospital, Rabat, Morocco; 2 Department of Orthopaedics, Chambéry Hospital, Chambéry, France

**Keywords:** Ligament triangulaire, instabilité luno-triquétrale, extenseur ulnaire du carpe, Triangular ligament, lunotriquetral instability, extensor carpi ulnaris

## Image en médecine

La dissociation luno-triquétrale fait partie des lésions du complexe triangulaire ; le diagnostic est évoqué cliniquement par des douleurs accentuées par l’inclinaison ulnaire et confirmé par l’arthroscanner ou l’IRM. Le traitement fait appel aux sutures arthroscopiques ou aux procédés de ligamentoplastie pour les lésions irréparables. La ligamentoplastie utilisant le transplant de l’extenseur ulnaire du carpe est une technique simple et séduisante pour le traitement de ces instabilités. Les auteurs rapportent l’observation d’un patient âgé de 40 ans, gaucher et banquier. Victime d’un accident de circulation occasionnant traumatismes de l’épaule et du poignet gauche. Le bilan lésionnel avait montré une fracture-luxation de l’épaule traitée orthopédiquement, associée à une entorse du poignet gauche. Une IRM du poignet a été demandée et qui a montré une lésion du ligament triangulaire (A). Après abord dorso-ulnaire, centré sur la radio ulnaire distale, et exposition de l’interligne luno-triquétrale, la rupture a été confirmée (B); ensuite, nous avons procédé à un avivement intra-carpien et mise en place de deux implants mini Mitek^®^ (B); le 3^ème^ temps opératoire a consisté en un prélèvement du transplant tendineux au dépens de l'extenseur ulnaire du carpe laissé pédiculé en distal (B); et enfin, le tendon a été fixé sur le lunatum et le triquétrum (B). Trois broches luno-triquétrales pour protéger la ligamentoplastie ont été mises en place (C), puis enlevées deux mois plus tard. L’évolution clinico-radiologique a été très bonne après six mois.

**Figure 1:**
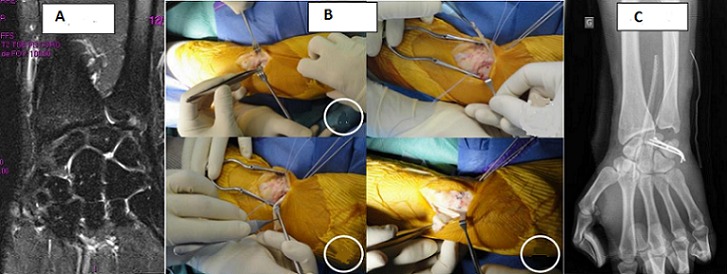
A) MRI of the wirst showing TFCC lesion; B) operative technique of lunotriquetral instability repair; C) three lunotriquetral K wires protecting the ligamentoplasty

